# ECPred: a tool for the prediction of the enzymatic functions of protein sequences based on the EC nomenclature

**DOI:** 10.1186/s12859-018-2368-y

**Published:** 2018-09-21

**Authors:** Alperen Dalkiran, Ahmet Sureyya Rifaioglu, Maria Jesus Martin, Rengul Cetin-Atalay, Volkan Atalay, Tunca Doğan

**Affiliations:** 1 Department of Computer Engineering, Middle East Technical University, 06800 Ankara, Turkey; 2 Department of Computer Engineering, Adana Science and Technology University, 01250 Adana, Turkey; 3 Department of Computer Engineering, Iskenderun Technical University, Hatay, 31200 İskenderun, Turkey; 4 European Molecular Biology Laboratory, European Bioinformatics Institute (EMBL-EBI), Hinxton, Cambridge, CB10 1SD UK; 5 KanSiL, Graduate School of Informatics, Middle East Technical University, 06800 Ankara, Turkey; 6 Graduate School of Informatics, Middle East Technical University, 06800 Ankara, Turkey

**Keywords:** Protein sequence, EC numbers, Function prediction, Machine learning, Benchmark datasets

## Abstract

**Background:**

The automated prediction of the enzymatic functions of uncharacterized proteins is a crucial topic in bioinformatics. Although several methods and tools have been proposed to classify enzymes, most of these studies are limited to specific functional classes and levels of the Enzyme Commission (EC) number hierarchy. Besides, most of the previous methods incorporated only a single input feature type, which limits the applicability to the wide functional space. Here, we proposed a novel enzymatic function prediction tool, ECPred, based on ensemble of machine learning classifiers.

**Results:**

In ECPred, each EC number constituted an individual class and therefore, had an independent learning model. Enzyme vs. non-enzyme classification is incorporated into ECPred along with a hierarchical prediction approach exploiting the tree structure of the EC nomenclature. ECPred provides predictions for 858 EC numbers in total including 6 main classes, 55 subclass classes, 163 sub-subclass classes and 634 substrate classes. The proposed method is tested and compared with the state-of-the-art enzyme function prediction tools by using independent temporal hold-out and no-Pfam datasets constructed during this study.

**Conclusions:**

ECPred is presented both as a stand-alone and a web based tool to provide probabilistic enzymatic function predictions (at all five levels of EC) for uncharacterized protein sequences. Also, the datasets of this study will be a valuable resource for future benchmarking studies. ECPred is available for download, together with all of the datasets used in this study, at: https://github.com/cansyl/ECPred. ECPred webserver can be accessed through http://cansyl.metu.edu.tr/ECPred.html.

## Background

Nomenclature Committee of the International Union of Biochemistry classifies enzymes according to the reactions they catalyse. Enzyme Commission (EC) numbers constitute an ontological system with the purpose of defining, organizing and storing enzyme functions in a curator friendly and machine readable format. Each EC number is a four digit numerical representation, four elements separated by periods (e.g., EC 3.1.3.16 - Protein-serine/threonine phosphatase), computationally stored within a unique ontology term. Four levels of EC numbers are related to each other in a functional hierarchy. Within the first level, the system annotates the main enzymatic classes (i.e., 1: oxidoreductases, 2: transferases, 3: hydrolases, 4: lyases, 5: isomerases and 6: ligases). The first digit in any EC number indicates which of the six main classes the annotated enzyme belongs to, the second digit represents the subclass class, the third digit expresses the sub-subclass class and the fourth digit shows the substrate of the enzyme [1]. Currently, the EC system is the universally accepted way of annotating the enzymes in biological databases.

Automated prediction of the enzymatic functions of uncharacterized proteins is an important topic in in the field of bioinformatics, due to both the high costs and the time-consuming nature of wet-lab based functional identification procedures. The hierarchical structure of EC nomenclature is suitable for automated function prediction. Several methods and tools have been proposed to classify enzymes [2–25]. However, most of the studies are limited to specific functional classes or to specific levels of the EC hierarchy, and there is limited availability considering methods that assume a top-down approach to classify all enzymatic levels (i.e. Level 0: enzyme or non-enzyme, Level 1: main class, Level 2: subclass, Level 3: sub-subclass and Level 4: substrate classes). One of the basic problems in this field is predicting whether an uncharacterized protein is an enzyme or not, and this topic is not considered in many previous studies. Besides, most of the previous methods incorporated only a single input feature type, which limits the applicability to the wide functional space. Furthermore, most of these previous tools are no longer available. Apart from the EC numbers, there are also other systems, such as the Gene Ontology (GO), an ontology that annotate the attributes of not only enzymes but also all other gene/protein families with molecular functions, cellular locations and large scale biological processes [26]. There are several automated methods that use GO to predict the functions of proteins including the enzymes [27–30].

In order to predict the functions of enzymes using classification methods the input samples (i.e., proteins) should be represented as quantitative vectors, reflecting their physical, chemical and biological properties. These representations are called feature vectors in the machine learning terminology. The selection of the type of representation is an important factor, which directly affects the predictive performance. Various types of protein feature representations have been proposed in the literature, and the major ones employed for the prediction of enzymatic functions can be categorized as homology [2, 10, 17], physicochemical properties [9], amino acid sequence-based properties [2, 3, 5, 7, 12, 14, 15, 21–25], and structural properties [6, 10, 11, 13, 18–20]. There are also a few EC number prediction methods, which utilize the chemical structural properties of compounds that interact with the enzymes [4, 13]. Finally, there are ensemble protein function prediction methods that integrate multiple types of protein feature representations at the input level in order to exploit the advantages of each [27–29]. The utilization of some of the feature types listed above (e.g., 3-D structural properties) require the characterization of proteins, which is a difficult and expensive procedure. Thus, only a sub-group of the proteins available in biological databases can be employed in these methods, which reduces the coverage of the predictions on the functional space. The second important factor in automated function prediction is the employed machine learning classification algorithm. The choice of algorithm, in relation to the data at hand, affects both the predictive performance and the computational complexity of the operation. In this sense, traditional and conventional classifiers such as the naïve Bayes classifier [20], *k* nearest neighbor classifier (*k*NN) [6, 11, 22, 24, 25], support vector machines (SVM) [5–7, 13, 14, 19, 21, 23], random forests (RF) [2, 4, 9], artificial neural networks (ANN) [3, 12], and only recently, deep neural networks (DNN) [16, 17] have been adapted for the problem of enzymatic function prediction. Many of these studies left out Level 0 prediction and focused mostly on EC Level 1. One of the most important criterion to evaluate an automated prediction system is the predictive performance. Many studies mentioned above reported performance values assessed based on their training accuracy (the reported rates are generally above 90 %.), which usually is not a good indicator due to the risk of overfitting. Here, we will focus on five studies, with which we compared the proposed method (i.e., ECPred): ProtFun, EzyPred, EFICAz, DEEPre, and COFACTOR.

Jensen et al. [3] proposed ProtFun, one of the first systems to perform enzyme function prediction using ANNs. In terms of the input feature types, post-translational modifications and localization features such as subcellular location, secondary structure and low complexity regions have been used in this method. ProtFun produces enzymatic function prediction on Level 0 and Level 1.

Shen and Chou [22] developed a web-based tool, EzyPred to predict the Level 0, Level 1 and Level 2 of the EC hierarchy using a top-down approach. Functional domain information was used to construct pseudo position-specific scoring matrices (Pse-PSSM) to be used as the input features. The optimized evidence-theoretic *k*-nearest neighbor (OET-*k*NN) algorithm was employed as the classifier, which was previously applied to the subcellular localization prediction problem.

EFICAz (the new version: EFICAz^2.5^) [10] is a webserver, which predicts EC number of protein sequences using a combination of approaches. EFICAz^2.5^ combines 6 different methods including CHIEFc (i.e., Conservation-controlled HMM Iterative procedure for Enzyme Family classification) family and multiple PFAM based functionally discriminating residue (FDR) identification, CHIEFc SIT evaluation, high-specificity multiple PROSITE pattern identification, CHIEFc and multiple PFAM family based SVM evaluation. EFICAz gives a complete four digit EC number prediction for a given target sequence. EFICAz is dependent on finding pre-defined domain or family signatures of the query sequences.

DEEPre [17] is a sequence-based EC number prediction method with a webserver, which employs deep neural networks as its classifier. Instead of using conventional types of features, DEEPre uses raw protein sequence based on two different types of encoding, sequence length dependent ones such as the amino acid sequence one-hot encoding, solvent accessibilities, secondary structures and position specific scoring matrices (PSSM), and sequence length independent ones, such as functional domain based encoding. Using these input features, convolutional neural network (CNN) and recurrent neural network (RNN) based deep learning classifier has been constructed. DEEPre predicts enzymatic functions on all levels of EC.

COFACTOR [18] is a protein function prediction webserver, which uses structural properties of proteins to predict Gene Ontology (GO) terms, EC numbers and ligand-binding sites. In the COFACTOR pipeline, first, the target protein structure is aligned with the template library. A confidence score is then calculated, based on both the global and local similarities between the target structure and template structures to assign the EC number of the most similar template enzyme to the target protein.

The objective in ECPred is to address all of the problems listed above and to generate a straightforward predictive method to be used in the fields of protein science and systems biology that works both as a web-based tool and as a stand-alone program through the command-line interface. While composing ECPred, a machine learning approach was pursued and multiple binary classifiers were constructed, each correspond to a specific enzymatic function (i.e. individual EC number). ECPred system was trained using the EC number annotations of characterized enzymes in the UniProtKB/Swiss-Prot database [31]. We developed a method for the construction of negative training datasets to reduce the number of potential false negatives in the training datasets. Positive and negative prediction score cut-off (i.e., threshold) values were individually determined for each classifier. The performance of ECPred was tested via cross-validation and with multiple independent test datasets and compared with the state-of-art methods in the field of enzyme classification. Finally, we built a web based service and a stand-alone tool by incorporating our models in a hierarchical manner.

## Implementation

### System design

In ECPred, each EC number constitutes an individual class and therefore, has an independent learning model. This brings the necessity of a separate model training for each EC number, with individual parameters (i.e., prediction score cut-offs), which are explained in Section “Class specific positive and negative score cut-offs”. ECPred was constructed considering an ensemble prediction approach, where the results of 3 different predictors (i.e., classifiers) with different qualities are combined. The machine learning-based predictors of ECPred are explained in Section “Predictors of ECPred”. The positive training dataset for an EC number is constructed using proteins that are annotated with that EC number in the UniProtKB/Swiss-Prot database. The negative training dataset for the same EC number is constructed by using both the proteins that have not been annotated with any enzymatic function (i.e. non-enzymes) and the proteins that are annotated with other EC numbers (i.e. proteins from different enzymatic families). The detailed procedure of negative training dataset construction is given in Section “Negative Training Dataset Generation Procedure” and the finalized training and validation dataset statistics are given in Section “Training and Validation Dataset Generation Rules and Statistics”. EC numbers which have more than 50 protein associations were chosen for training by ECPred, for statistical power. Totally, 858 EC classes (including 6 main class, 55 subclass, 163 sub-subclass and 634 substrate EC numbers), satisfied this condition, and thus trained under the ECPred system.

ECPred first predicts whether a query sequence is an enzyme or a non-enzyme, together with the prediction of the main EC class (in the case that the query is predicted to be an enzyme). After deciding the main EC class of a query, subclass, sub-subclass and substrate classes are predicted. The flow-chart of ECPred along with the prediction route for an example query is given in Section “Prediction procedure”.

### Predictors of ECPred

ECPred combines three independent predictors: SPMap, BLAST-*k*NN and Pepstats-SVM that are based on subsequences, sequence similarities, and amino acid physicochemical features, respectively. The ensemble-based methodology used here is explained in our previous publications, where we constructed a protein function prediction tool using Gene Ontology terms [29, 32, 33]. The training procedure of the individual predictors are briefly explained below.

#### SPMap

Sarac et al. [32] developed a subsequence-based method called Subsequence Profile Map (SPMap), to predict protein functions. SPMap consists of two main parts: subsequence profile map construction and feature vector generation. Subsequence profile map construction part further consists of three modules: subsequence extraction module, clustering module and probabilistic profile construction module. In the subsequence extraction module, all possible subsequences for given length *l* are extracted from the positive training dataset using the sliding window technique. After that, the subsequences are clustered in the clustering module, based on their pairwise similarities. Blocks substitution matrix (BLOSUM62) [34] is used to calculate the similarity score between two subsequences via a simple string comparison procedure. At a given instant of time, a subsequence is compared with the subsequences in all existing clusters and assigned to the cluster which gives the highest similarity score. Similarity score *s(x, y)* between two subsequences is calculated as follows.

1 $$ s\left(x,y\right)=\sum \limits_{i=1}^lM\left(x(i),y(i)\right) $$

where *x(i)* i s the amino acid at the *i*^*th*^ position of the subsequence *x* and *M(x(i), y(i))* is the similarity score in BLOSUM62 matrix for the *i*^*th*^ position of *x* and *y*. After calculating similarity score between a cluster *c* and a subsequence *ss*, if *s* (*c*, *ss*) ≥ *t* (*t* denotes the similarity score threshold), the subsequence *ss* is assigned to *c*; otherwise a new cluster is generated. The threshold value (*t*) used here is 8, the selection of which was discussed in our previous paper [32]. After all clusters are generated, a position specific scoring matrix (PSSM) is created for each cluster in the probabilistic profile construction module. PSSMs consist of *l* columns and 20 rows (amino acids). The amino acid count for each position is stored in the PSSM and the value of each matrix element is determined by the amino acid count of the subsequences assigned to that cluster. Subsequently, each PSSM is converted to a probabilistic profile. Let *S*_*c*_ denote the total number of subsequences in cluster *c*. If *S*_*c*_ is less than 10% of the positive training dataset size, that cluster is discarded; otherwise, a probabilistic profile is generated. The reason behind this application is that the discarded clusters’ PSSMs would only hit a few sequences, resulting in a scarcely populated dimension on the feature vectors, and thus have an insignificant contribution to the classification. Let the amino acid count for the amino acid *j* at the *i*^*th*^ position of the subsequence be shown by *aa*_*count*_*(i, j),* the probability of the amino acid *j* to occur at the *i*^*th*^ position of the subsequence: *PP*_*c*_*(i, j)* is then calculated as follows.

2 $$ {PP}_c\left(i,j\right)=\mathit{\log}\frac{aa_{count}+0.01}{S_c} $$

*0.01* is added to the amino acid count for each position to avoid zero probabilities. Next, feature vectors (each correspond to an individual query sequence) are generated by using the subsequences of the query sequences and the extracted probabilistic profiles. The size of the feature vector is the same as the number of probabilistic profiles (i.e., the number of clusters). Here, we consider the highest probability value when assigning a query subsequence to a profile. In a more formal definition, each subsequence *ss* is first compared with a probabilistic profile *PP*_*c*_ and a probability is computed as:

3 $$ P\left( ss|{PP}_c\right)=\sum \limits_{i=1}^l{PP}_c\left(i, ss(i)\right) $$

The *c*^*th*^ dimension element of the feature vector *V* is then determined as follows:

4 $$ V(c)=\underset{ss\in E}{\mathit{\max}}P\left( ss|{PP}_c\right) $$

where the probability value of the subsequence *ss* of protein *E* with the highest probability on *PP*_*c*_ is assigned to the *c*^*th*^ element of the feature vector. After that, the elements of the feature vector are changed back to natural logarithms (between 0 and 1), using exponential function. The same operations are applied for the proteins in both the positive and negative datasets, and finally, a training file is created. Support vector machines (SVM) classifier is then used for the classification.

#### BLAST-*k*NN

In order to classify a target protein, the *k*-nearest neighbor algorithm is used, where the similarities between the query protein and proteins in the training dataset are calculated using the NCBI-BLAST tool [35]. *k*-nearest neighbors with the highest BLAST scores are extracted. The output *O*_*B*_ of BLAST-*k*NN, for a query protein, is calculated as follows:

5 $$ {O}_B=\frac{S_p-{S}_n}{S_p+{S}_n} $$

where *S*_*p*_ is the sum of the BLAST scores of proteins in the *k*-nearest neighbors in the positive training dataset. Similarly, *S*_*n*_ is the sum of scores of the *k*-nearest neighbor proteins in the negative training dataset. Note that the value of *O*_*B*_ is between − 1 and + 1. The output is 1 if all *k* nearest proteins are elements of the positive training dataset and − 1 if all *k* proteins are from the negative training dataset. In BLAST-*k*NN, *O*_*B*_ is directly used as the prediction score.

#### Pepstats-SVM

The Pepstats tool [36] is a part of European Molecular Biology Open Software Suite (EMBOSS), and constructed to extract the peptide statistics of the proteins (e.g., molecular weight, isoelectric point, physicochemical properties and etc.). In Pepstats, each protein is represented by a 37-dimensional vector. These features are scaled and subsequently fed to the SVM classifier [37] as input.

**Fig. 1:**
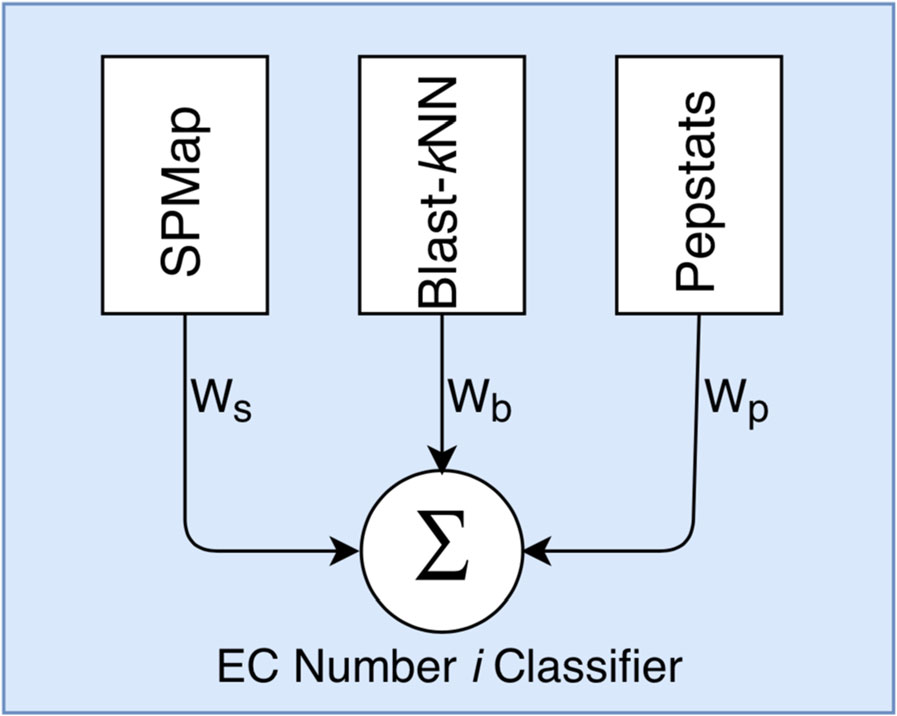
Structure of an EC number classifier in ECPred

For a query protein sequence, ECPred combines the individual prediction scores of these three predictors (shown in Fig. 1). A 5-fold cross-validation is applied for each method and the area under the receiver operating characteristic curve (AUROC) is calculated for BLAST-*k*NN, Pepstats-SVM and SPMap, individually. Using these AUROC values, all three methods are combined and weighted mean score for each method is calculated [33]. The weight for method *m* where *m ϵ {BLAST-kNN; PEPSTSTATS-SVM; SPMap}* is calculated as follows;

6 $$ W(m)=\frac{R_m^4}{R_{BLAST- kNN}^4+{R}_{SPMap}^4+{R}_{PEPSTATS- SVM}^4} $$

where the weight of the method *m* is represented by *W(m)*. *R*_*m*_ stands for AUROC value for method *m*. The weights of the base predictors are calculated individually for each EC class model. When a query protein is given as input to ECPred, first it is run for base predictors individually (i.e., SPMap, Blast-*k*NN and Pepstats), each of which produces a prediction score to associate the query protein with the corresponding EC number. Then, these scores are multiplied with the class-specific weights and summed up to produce the weighted mean score, which corresponds to the finalized prediction score for the query protein for that EC number.

The approaches employed in each individual predictor has both advantages and disadvantages in predicting different enzymatic classes. For example, GDP binding domains of G-proteins has unique structural features which are well conserved, thus a homology-based approach that considers the overall sequence similarity would be effective in identifying these domains. Apart from that, proteins which are targeted to endoplasmic reticulum carry short signal peptides independent of their overall structure hence a subsequence-based approach would be more appropriate for these types of proteins. Each enzymatic function can be differentiated by different types of classifiers; therefore, their weighted combination achieves the best performance.

### Prediction procedure

**Fig. 2:**
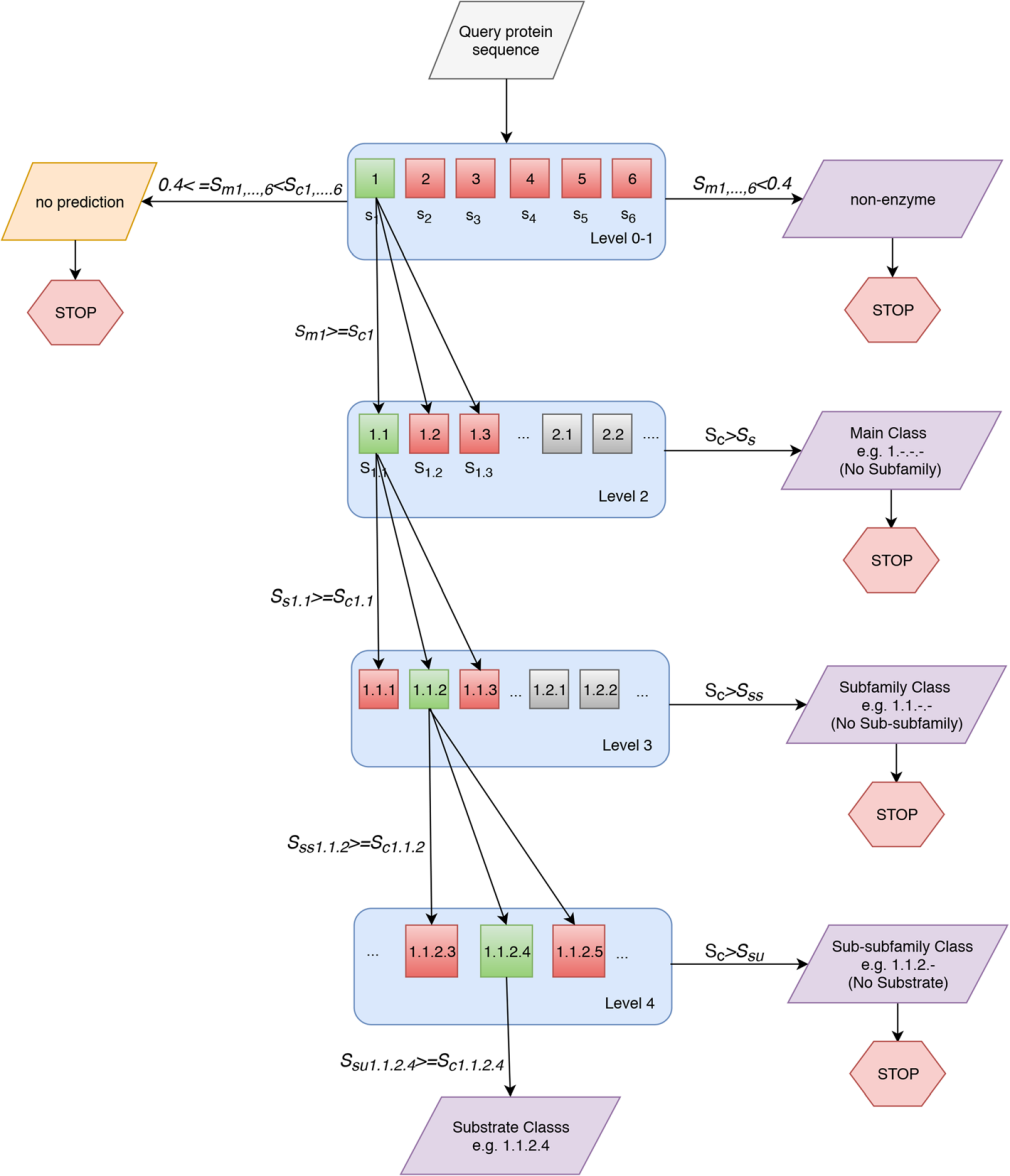
Flowchart of ECPred together with the prediction route of an example query protein. Query protein (*P*_*Q*_) received a score that is higher than the class specific positive cut-off value of main EC class 1.-.-.- (i.e., oxidoreductase) at Level 0–1 classification (*S*_*m1*_ > *S*_*c1*_); as a result, the query is only directed to the models for the subclasses of main class 1.-.-.-. Considering the subclass prediction (Level 2), *P*_*Q*_ received a high score (*S*_*s1.1*_ > *S*_*c1.1*_) for EC 1.1.-.- (i.e., acting on the CH-OH group of donors) and further directed to the children sub-subclass EC numbers, where it received a high score (*S*_*ss1.1.2*_ > *S*_*c1.1.2*_) for EC 1.1.2.- (i.e., with a cytochrome as acceptor) at Level 3, and another high score (*S*_*u1.1.2.4*_ > *S*_*c1.1.2.4*_) for EC 1.1.2.4 (i.e., D-lactate dehydrogenase - cytochrome) at the substrate level (Level 4) and received the final prediction of EC 1.1.2.4

The flowchart of the method is given in Fig. 2 together with a toy example where the tool produced the prediction EC 1.1.2.4 for the query. Given a query protein, the algorithm starts with the prediction of enzyme vs. non-enzyme (Level 0) together with main class (Level 1) predictions (i.e. 1.-.-.-, 2.-.-.-, 3.-.-.-, 4.-.-.-, 5.-.-.- or 6.-.-.-). After deciding the main EC class, subclass, sub-subclass and substrate classes of the query protein are predicted subsequently.

The rules of producing predictions are given below:

1. Main classes (i.e. Level 0 and Level 1):
  - If only one of the main classes obtains a prediction score over the class specific positive cut-off value, the query protein will receive the corresponding EC number as the prediction and the algorithm continues with the models for the descendants of that main class EC number;
  - if multiple classes produced higher-than-positive-cut-off scores for the query protein, the main class with the maximum prediction score will be given as the prediction and the algorithm continues with the models for the descendants of that main class EC number;
  - if the prediction score is lower than the pre-specified negative cut-off score for all main EC classes, algorithm stops and the query protein is labeled as a non-enzyme;
  - for the rest of the cases, algorithm stops as there will be no prediction for the query protein.
2. Subclasses, sub-subclasses, substrates (i.e., Level 2, Level 3 and Level 4):
  - If only one of the subclasses obtains a prediction score over the class specific positive cut-off value, the query protein will receive the corresponding EC number as prediction and the algorithm continues with the models for the descendants of the corresponding subclass EC number (if there are any);
  - if multiple subclasses produced higher-than-positive-cut-off scores for the query protein, the subclass with the maximum prediction score will be given as the prediction and the algorithm continues with the models for the descendants of the corresponding subclass EC number (if there are any);
  - if the prediction score is lower than the subclass specific positive cut-off values for all of the EC subclasses at that level, algorithm stops and the query protein receives the finalized label, which is the EC number prediction obtained from the previous level.

### Negative training dataset generation procedure

Since ECPred is composed of binary classifiers, positive and negative datasets are required for training. There is a basic problem in many existing studies related to the construction of negative datasets. The conventional procedure is to simply select all of the proteins that are not in the positive dataset as the negative dataset samples, for that class. In our case, this conventional procedure is translated as follows: if a protein is not annotated with a specific EC number, that protein could be included in the negative dataset for that EC class. However, this approach is problematic. These conventionally generated negative sets potentially include the proteins that actually have the corresponding function, but the annotation has not been yet recorded in the source database (i.e., false negatives). Such cases may lead to confusion for the classifier and thus may reduce the classification performance.

**Fig. 3:**
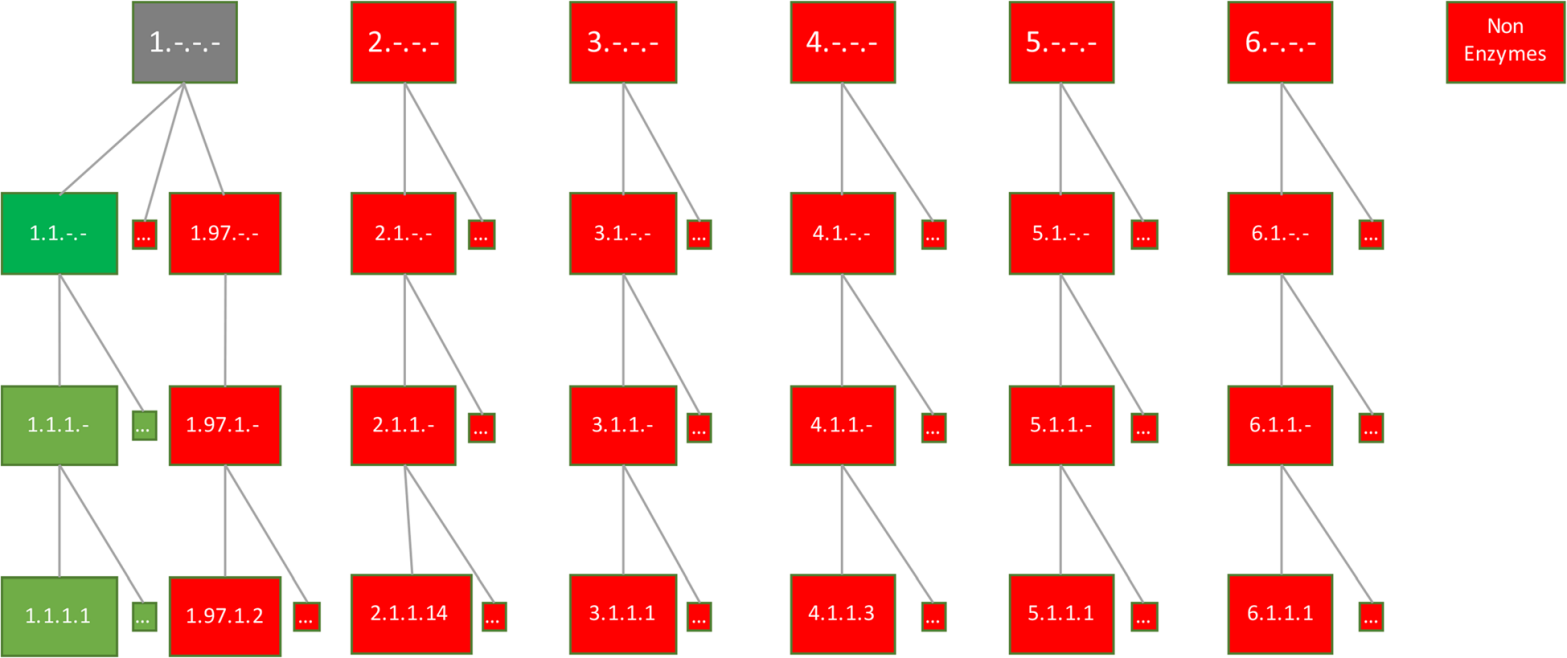
Positive and negative training dataset construction for EC class 1.1.-.-. Green colour indicates that the members of that class are used in the positive training dataset, grey colour indicates that the members of that class are used neither in the positive training dataset, nor in the negative training dataset and red colour indicates that the members of that class are used in the negative training dataset

In ECPred, a negative dataset is composed of two parts: *(i)* samples coming from other enzyme families, and *(ii)* the non-enzyme samples. In order to avoid including ambiguous samples in the negative datasets, we have developed a hierarchical approach to select negative training dataset instances for each EC class. Fig. 3 shows the positive and negative training dataset generation for the example EC class 1.1.-.-. Proteins annotated with EC 1.1.-.- and its children (e.g., 1.1.1.-, 1.1.2.-, …) are included in the positive training dataset (green coloured boxes); whereas, proteins annotated with siblings of 1.1.-.- (e.g., 1.2.-.-, 1.3.-.-, …) and children of these siblings (e.g., 1.2.1.-, 1.3.1.-, …), and all the proteins annotated with the other EC main classes together with their respective subclasses (e.g., 2.-.-.-, 3.-.-.-, … and their children terms) and selected non-enzymes are included in the negative training dataset for EC 1.1.-.- (red coloured boxes).

The selection of non-enzyme proteins for the negative training datasets required additional information. There is no specific annotation that marks sequences as non-enzymes in major protein resources. Therefore, we had to assume that proteins without a documented enzymatic activity should be non-enzymes. However, this assumption brings the abovementioned ambiguity about whether a protein is a true negative or a non-documented positive sample. In UniProtKB, each protein entry has an annotation score between 1 star to 5 star. An annotation score of 5 star indicates that the protein is well studied and reliably associated with functional terms, while the annotation score of 1 star means that the protein only has a basic annotation that is possibly missing a lot about its functional properties. We tried to make sure that only reliable non-enzymes are included in the negative dataset by selecting the proteins that have an annotation score of 4 or 5 stars and without any enzymatic function annotation. By constructing the negative datasets with these rules, we also tried to include a wide selection of proteins, covering most of the negative functional space; as well as, excluding ambiguous cases.

### Training and validation dataset generation rules and statistics

In this section, we focused on training and validation datasets while the test datasets are described in the Results and Discussion section. Protein sequences and their EC Number annotations are taken from UniProtKB/Swiss-Prot (release: 2017_3). All proteins that are associated with any of the EC numbers were initially downloaded from the database (approximately 248,000 protein entries) and proteins that are associated with more than one EC number (approximately 0.5% of all enzyme entries) were discarded, since multi-functional enzymes may be confusing for the classifiers. After that, all annotations were propagated to the parents of the annotated EC number, according to the EC system’s inheritance relationship. Finally, EC classes that are associated with at least 50 proteins were selected for the training. Totally, 858 EC classes (including 6 main EC classes) satisfied this condition. Table 1 shows the statistics of the initial datasets (second column) for each EC main class, together with the non-enzyme proteins that satisfied the conditions explained above. The third column indicates the number UniRef50 clusters [38] for the protein datasets given in the preceding column. In UniRef50, sequences that are greater than or equal to 50% similar to each other are clustered together; so that, the value in this column indicates how diverse the enzymes in a particular EC main class are.

**Table 1:** The number of proteins and UniRef50 clusters in the initial dataset for each main enzyme class and for non-enzymes

EC main classes	# of proteins	# of UniRef50 clusters
Oxidoreductases	36,577	8242
Transferases	86,163	20,133
Hydrolases	59,551	16,018
Lyases	22,368	3475
Isomerases	13,615	2883
Ligases	29,233	4429
Non-Enzyme	42,382	25,333

Instead of directly using all of the proteins (shown in the second column of Table 1) for system training and validation with random separation, we chose the representative protein entries from the corresponding UniRef50 clusters and employed and randomly separated this set for training and validation (90% to 10% distribution). This way, sequences that are very similar to each other would not end up both in training and validation datasets, which would otherwise cause model overfitting and the overestimation of the system performance. The final configuration of the training and validation datasets are given in Table 2. 10% of the UniRef50 clusters were used for the positive validation dataset and 90% was employed for the positive training dataset. These same separation ratio was used for the negative validation and the negative training datasets. For each class, the enzyme part of the negative training dataset was constructed using the proteins from the other five main enzyme classes. The number of proteins in the negative training datasets were fixed to make them equal to the number of proteins in the positive datasets, to obtain balanced training datasets. A similar procedure was applied to generate the datasets of the EC numbers at the subclass, sub-subclass and substrate levels.

**Table 2:** The number of proteins that were used in the training and validation of ECPred, for each main enzyme class

EC main classes	Positive Training Dataset Size	Negative Training Dataset Size		Positive Validation Dataset Size	Negative Validation Dataset Size
		Enzymes^a^	Non-enzymes		
Oxidoreductases	7417	3709	3709	825	822
Transferases	18,119	9060	9060	2014	2012
Hydrolases	14,416	7208	7208	1602	1601
Lyases	3127	1564	1564	348	344
Isomerases	2549	1275	1275	284	282
Ligases	3986	1993	1993	443	441

^a^Equal number of enzymes were selected from the other EC classes

### Class specific positive and negative score cut-offs

Positive and negative optimal score cut-off values were calculated for each EC class, in order to generate binary predictions from continuous score values. The cut-off values were determined during the cross-validation procedure. For any arbitrarily selected score cut-off value, if a protein from the positive validation dataset obtained a prediction score above the cut-off value, it was labeled as a true positive (TP); otherwise, it was labeled as a false negative (FN). Furthermore, if a protein from the negative validation dataset got a prediction score above the score cut-off value, it was labeled as a false positive (FP); otherwise, it was labeled as a true negative (TN). After determining all TPs, FPs, FNs and TNs; precision, recall and F1-score values were calculated. This procedure was repeated for all arbitrarily selected score cut-off values. The cut-off value, which provided the highest classification performance in terms of F1-score was selected as the positive cut-off value for that EC number class. A similar procedure was pursued to select the negative score cut-off values. After investigating the automatically selected negative cut-off values for all EC number classes, we observed that highest F1-scores were obtained for the values around 0.3; therefore, we decided to select 0.3 as the global negative score cut-off value for all classes. The positive cut-off values varied between 0.5 to 0.9. The reason behind selecting two different cut-off values for the negative and positive predictions was to leave the ambiguous cases without any prediction decision (i.e. no prediction).

## Results and discussion

### ECPred validation performance analysis

The overall predictive performance of each EC number class was measured on the class-specific validation datasets, the generation of which were explained in the Methods section. The average level specific performance results in terms of precision (i.e., *TP / TP + FP*), recall (i.e., *TP / TP + FN*) and F1-score (i.e., the harmonic mean of precision and recall) values are shown in Table 3. The performance in the validation analysis was considerably high (i.e., below 0.90 for only 11 EC numbers). UniRef50 cluster were employed in the validation analysis in order to separate the training and validation instances from each other with at least 50% sequence divergence; so that the results would not be biased. However, sequence similarity was still an important factor, which might led to the overestimation of the performance. In order to observe better estimates of the performance of ECPred, we carried out additional analyses using independent test sets, which are explained in the following sub-sections. In general, the validation performance results indicated that ECPred can be a good alternative to predict the enzymatic functions of fully uncharacterized proteins, where the only available information is the amino acid sequence.

**Table 3:** The performance results of the ECPred validation analysis

EC Level	F1-score	Recall	Precision
Level 0	0.96	0.96	0.96
Level 1	0.96	0.96	0.96
Level 2	0.98	0.97	0.99
Level 3	0.99	0.98	0.99
Level 4	0.99	0.99	0.99

### Performance comparison with the state-of-the-art tools via independent test sets

#### Temporal hold-out dataset test

An independent time-separated hold-out test dataset was constructed in order to measure the performance of ECPred and to compare it with the existing EC number prediction tools. This dataset consisted of 30 proteins that did not have any EC number annotation at the time of ECPred system training (UniProtKB/Swiss-Prot release 2017_3), but annotated with an EC number in UniProtKB/Swiss-Prot release 2017_6; and another 30 proteins still without an EC number annotation (i.e., non-enzymes) that have an annotation score of 5. These 60 proteins were never used in the ECPred system training. The UniProt accession list of the temporal hold-out test dataset proteins are given in the ECPred repository. These proteins were fed to ProtFun, EzyPred, EFICAz, DEEPre tools along with ECPred, and the resulting predictions were compared to the true EC number labels of these proteins to calculate the predictive performances. All compared methods were run in default settings, as given in both their respective papers and web servers. Tables 4, 5, 6 and 7 display the performance results for Level 0, Level 1, Level 2, and Level 3 EC classes, respectively. In these tables, the best performances are highlighted in bold fonts. In Tables 6 and 7, the state-of-the-art prediction tools, which do not predict EC numbers at those respective levels, are not shown. The substrate level EC number prediction performances are not given in a table because the compared tools produced zero performance on this level. ECPred performed with F1-score = 0.14, recall = 0.10 and precision = 0.21 on the substrate level. It is important to note that, some resources consider the prediction of the substrate level EC numbers unreliable [17].

**Table 4:** Temporal hold-out test enzyme – non-enzyme (Level 0) prediction performance comparison

Method	F1-score	Recall	Precision
ProtFun	0.79	0.87	0.72
EzyPred	0.15	0.13	0.16
EFICAz	0.42	0.30	0.69
DEEPre	0.53	0.43	0.68
ECPred-wne	0.65	0.93	0.50
ECPred	**0.83**	**0.97**	**0.73**

**Table 5:** Temporal hold-out test EC main class (Level 1) prediction performance comparison

Method	F1-score	Recall	Precision
ProtFun	0.12	0.10	0.15
EzyPred	0.15	0.13	0.16
EFICAz	0.42	0.30	**0.69**
DEEPre	**0.50**	0.40	0.67
ECPred-wne	0.40	**0.48**	0.34
ECPred	0.48	**0.43**	0.54

**Table 6:** Temporal hold-out test EC subclass class (Level 2) prediction performance comparison

Method	F1-score	Recall	Precision
EzyPred	0.11	0.10	0.13
EFICAz	0.11	0.07	0.33
DEEPre	0.11	**0.25**	0.07
ECPred	**0.26**	0.20	**0.35**

**Table 7:** Temporal hold-out test EC sub-subclass class (Level 3) prediction performance comparison

Method	F1-score	Recall	Precision
DEEPre	0.05	0.03	0.14
ECPred	**0.22**	**0.17**	**0.31**

There are two observations from Tables 4, 5, 6 and 7; first of all, the predictive performance significantly decreases with the increasing EC levels, for all methods. The probable reason is that, the number of training instances diminishes going from generic to specific EC numbers, which is crucial for proper predictive system training. This is more evident for DEEPre (please refer to Tables 6 and 7), which employs deep neural networks (DNN) as its classification algorithm, as DNNs generally require higher number of training instances compared to the conventional machine learning classifiers. The second observation from Tables 4, 5, 6 and 7 is that, ECPred performed as the best classifier in most cases and produced comparable results for the rest, indicating the effectiveness of the proposed methodology in enzyme function prediction. It was also observed that ECPred was more robust against the problem of low number of training instances. At Level 1 prediction, ECPred and DEEPre performances were very close but on the higher levels ECPred performed better. The better performance of ECPred at high EC levels can be attributed to the employed straightforward methodology, where independent binary classifiers are used for all EC number classes. It is also important to note that, the performance values of the state-of-the-art methods given here can be significantly different from the values given in the original publications of these methods. The reason behind this is that, the test samples we used here are extremely difficult cases for predictors. Most of the enzymes in the temporal hold-out set have low number of homologous sequences in the database of known enzymes, which also is one of the reasons that these proteins were not annotated as enzymes in the source databases before. We believe our test sets reflect the real world situation better, where automated predictors are expected to annotate uncharacterized proteins without well annotated homologs.

At this point in the study, we tested the effectiveness of the proposed negative training dataset construction approach. For this, the six main EC class models have been re-trained without the incorporation of the non-enzyme sequences in the negative training datasets. Instead, the negative training dataset of a main EC class model only included enzymes from the other five main EC classes. This variant of ECPred is called ECPred-wne (ECPred without non enzymes). We tested the performance of ECPred-wne using the temporal hold-out test set. The results of this test are shown in the Tables 4 and 5, where it is observed that the performance of ECPred decreases significantly without the involvement of the non-enzyme sequences, indicating the effectives of the negative training dataset construction approach proposed here.

##### Classifier comparison on the temporal hold-out dataset test

In order to observe the performance of the individual predictors incorporated in ECPred (and to compare them with their weighted mean - the finalized ECPred) we carried out another test using the temporal hold-out set, where we calculated the predictive performance of BLAST-*k*nn, SPMap and Pepstats-SVM individually. As shown in Tables 8 and 9, ECPred performed as good as the best individual predictor for the level 0 prediction (i.e., Pepstats-SVM). Also, ECPred performed slightly better compared to the best individual predictor in the main class prediction task (i.e., BLAST-*k*nn). Pepstats-SVM is a tool based on the physiochemical properties of amino acids and their statistics found in the protein sequences. Enzyme and non-enzyme classes can be differentiated by this property, since enzymes have preferences on certain types of functional residues such as the polar and hydrophilic amino acids. Therefore, Pepstats-SVM performs better in differentiating enzymes from non-enzymes. When we consider the main EC classes, BLAST-*k*NN performs better, since there are certain motifs in the active regions of enzymes, which can be captured by the BLAST-*k*NN. On overall, ECPred performs either as good as or better than the individual predictors at each EC level by calculating their weighted mean.

**Table 8:** Performance comparison of the individual predictors and ECPred for enzyme – non-enzyme (level 0) prediction

Method	F1-score	Recall	Precision
SPMap	0.82	0.90	**0.75**
BLAST-*k*NN	0.75	0.83	0.68
Pepstats-SVM	**0.83**	**0.97**	0.73
ECPred	**0.83**	**0.97**	0.73

**Table 9:** Performance comparison of the individual predictors and ECPred for the main EC class (level 1) prediction

Method	F1-score	Recall	Precision
SPMap	0.23	0.17	0.36
BLAST-*k*NN	0.47	**0.43**	0.52
Pepstats-SVM	0.26	0.20	0.35
ECPred	**0.48**	**0.43**	**0.54**

#### No domain annotation dataset test

Protein Families Database (Pfam) [39] uses functional domain information to assign EC numbers to protein sequences. Since structural domains are the evolutionary and functional units in proteins, it is logical to associate enzymatic functions (through EC numbers) to protein domains. Sophisticated domain annotation algorithms predict the presence of these domains on uncharacterized protein sequences. This way, large-scale automated enzyme function predictions are produced. However, there is still need for novel predictive methods to produce enzymatic function annotations for the proteins without any domain annotation. In order to investigate ECPred’s ability to predict functions of enzymes which don’t have domain information, a dataset called no-Pfam test, which consists of 40 enzymes and 48 non-enzymes, was constructed. The proteins in this dataset were not used during the training of ECPred. The UniProt accession list of the no-Pfam test dataset proteins are given in the ECPred repository. These proteins were fed to EzyPred, EFICAz, DEEPre tools along with ECPred, and the resulting predictions were compared to the true EC number labels of these proteins to calculate the predictive performances. Tables 10, 11, 12, 13 and 14 display the performance results for Level 0, Level 1, Level 2, Level 3 and Level 4 EC classes, respectively. In these tables, the best performances are highlighted in bold fonts. The results show that, ECPred can predict the enzymatic functions of proteins with no Pfam domain information. On no-Pfam test set, the performance difference between ECPred and the next best performer DEEPre is more evident even on Level 1 prediction (please refer to Tables 5 and 9). One possible reason is that, DEEPre uses domain annotation as one of its input feature types; on the other hand, ECPred only takes amino acid sequences as its input. Another important observation here is that, ECPred performed significantly better in Level 0 prediction, both on the temporal hold-out test dataset and on the no-Pfam test set. This ability is attributed to the sophisticated negative training dataset construction method proposed here, where the negative datasets covered a high portion of the functional space.

**Table 10:** No-Pfam test dataset enzyme – non-enzyme (Level 0) prediction performance comparison

Methods	F1-score	Recall	Precision
EzyPred	0.54	0.54	0.54
EFICAz	0.37	0.23	**1.00**
DEEPre	0.60	0.4	0.85
ECPred	**0.85**	**0.82**	0.89

**Table 11:** No-Pfam test dataset EC main class (Level 1) prediction performance comparison

Methods	F1-score	Recall	Precision
EzyPred	0.42	0.39	0.46
EFICAz	0.33	0.20	**1.00**
DEEPre	0.52	0.38	0.82
ECPred	**0.73**	**0.63**	0.86

**Table 12:** No-Pfam test dataset EC subclass class (Level 2) prediction performance comparison

Methods	F1-score	Recall	Precision
EzyPred	0.30	0.26	0.36
EFICAz	0.33	0.20	**1.00**
DEEPre	0.40	0.27	0.77
ECPred	**0.60**	**0.47**	0.82

**Table 13:** No-Pfam test dataset EC sub-subclass class (Level 3) prediction performance comparison

Methods	F-score	Recall	Precision
EFICAz	0.33	0.20	**1.00**
DEEPre	0.33	0.22	0.73
ECPred	**0.58**	**0.45**	0.81

**Table 14:** No-Pfam test dataset EC substrate class (Level 4) prediction performance comparison

Methods	F-score	Recall	Precision
EFICAz	0.33	0.20	**1.00**
DEEPre	0.33	0.22	0.73
ECPred	**0.39**	**0.26**	0.71

#### COFACTOR dataset test

COFACTOR is a protein function prediction tool that use 3-D structural information of proteins [18, 40]. In their article, the authors of the COFACTOR tool constructed an independent test dataset [18], which was also employed in later studies with the purpose of benchmarking [17]. This dataset is composed of PDB ids and their corresponding amino acid sequences, instead of full protein sequences, and considered to be a difficult dataset for enzymatic function prediction. In this experiment, ECPred was ran on the COFACTOR test dataset, which consisted 318 samples. The same procedure was applied to produce predictions on the COFACTOR test dataset samples and the performance values were calculated according to the true EC number labels. ECPred obtained 0.95, 0.87 and 0.90 in terms of macro-precision, macro-recall and macro-F1, respectively on the COFACTOR test dataset EC main class (Level 1) prediction task. The detailed COFACTOR test dataset results of the state-of-the-art methods are given in Li et al. [17] together with the explanation of the performance measures. ECPred outperformed all methods under macro-precision and macro-F1 measures. It also outperformed all methods under macro-recall, except DEEPre, where both methods obtained similar results. On the subclass prediction task, ECPred obtained 0.92, 0.78 and 0.83 macro-precision, macro-recall and macro-F1, respectively. ECPred again outperformed all the other methods under macro-precision and macro-F1 measures in COFACTOR test dataset subclass prediction, and produced comparable results in terms of macro-recall [17].

Since all of the features incorporated in ECpred are sequence-based, it can predict the functions of completely uncharacterized enzymes without any known domain/family signatures or motifs, which is an advantage over methods that rely on existing annotation or ocumented signatures. The results of the tests indicated the effectiveness of the methodological approach used in ECPred, which is mainly intended to be employed to help experimental researchers to plan their further research and to aid expert curators in protein function annotation.

## Conclusions

ECPred is an automated EC number based enzymatic function prediction method, that takes the amino acid sequences as inputs. ECPred adopts a supervised ensemble classification approach by incorporating 3 different predictors based on homology, subsequence extraction and peptide physicochemical properties. We trained independent classification models for each EC number, which enabled the optimization of the parameters according to the respective enzymatic function. ECPred was trained and validated using the enzyme entries located in the UniProtKB/Swiss-Prot database. We rigorously tested and compared the proposed method with the state-of-the-art EC number prediction tools by constructing various independent test datasets and running ECPred and the other tools on them. The results of these analyses showed that ECPred is able to predict the enzymatic functions of uncharacterized proteins at all five levels of EC, and ECPred’s prediction performance was better compared to the other tools, in most of the test cases. All of the datasets employed in this study, together with the prediction results of ECPred are available in the ECPred repository (https://github.com/cansyl/ECPred/). Especially the test datasets constructed here (i.e., temporal hold-out and no-Pfam datasets) will be valuable for future studies, where they can be employed for benchmarking purposes.

ECPred was constructed as a Java based stand-alone tool that accepts FASTA protein sequence files containing up to 20 proteins, as inputs. The output is a “tsv” file containing the EC main class, subclass, sub-subclass and substrate class predictions together with a confidence score; alternatively, the output can be “non-enzyme” or “no prediction”. The detailed information regarding the download, installation and the usage of ECPred is provided at https://github.com/cansyl/ECPred/. Furthermore, an online webserver was constructed for ECPred to give EC number prediction for a given sequence. The webserver is available at http://cansyl.metu.edu.tr/ECPred.html.

## Availability and requirements

Project name: ECPred

Project home page: http://cansyl.metu.edu.tr/ECPred.html

Operating system(s): Mac OS X, Unix and Linux compatible stand-alone tool; and a platform independent web based tool

Programming language: Java

Other requirements: Java 1.3.1 or higher

License: GNU GPL

Any restrictions to use by non-academics: Restrictions are specified by GNU GPL v3

## Abbreviations

DNN: Deep neural network
EC: Enzyme Commission
